# Livelihoods of young women with and without disabilities in KwaZulu-Natal during COVID-19

**DOI:** 10.4102/ajod.v13i0.1400

**Published:** 2024-07-19

**Authors:** Jill Hanass-Hancock, Ayanda Nzuza, Samantha Willan, Thesandree Padayachee, Mercilene Machisa, Bradley Carpenter

**Affiliations:** 1 Gender and Health Research Unit, South African Medical Research Council, Durban, South Africa; 2 School of Health Science, University of KwaZulu-Natal, Durban, South Africa; 3 Gender and Health Research Unit, South African Medical Research Council, Pretoria, South Africa; 4 Gender and Health Research Unit, South African Medical Research Council, Cape Town, South Africa

**Keywords:** disability, livelihoods, COVID-19, South Africa, food security, crisis, pandemic

## Abstract

**Background:**

Persons with disabilities are more likely to have poorer livelihood outcomes, including food insecurity. Inequalities are heightened for young women with disabilities, especially in times of crisis.

**Objectives:**

To understand the livelihood experience of young South African women with and without disabilities during the coronavirus pandemic (COVID-19).

**Method:**

We conducted a longitudinal study with 72 young women with and without disabilities enrolled in tertiary institutions in eThekwini, South Africa. We undertook a series of in-depth interviews collecting quantitative and qualitative data, prompting participants’ experiences during the COVID-19 pandemic, including living arrangements, impact on education, access to resources and food security.

**Results:**

Participants reported livelihood changes related to living arrangements, education, income, and social connectedness during the pandemic. Social grants (old-age pension, child support, disability grant) and student stipends were critical financial resources to ensure food security. Participants with disabilities were more likely to experience food insecurities and moderate hunger, with their households having less access to mitigating resources such as land or livestock. Deaf participants also reported social isolation.

**Conclusion:**

The study shows that social protection mechanisms mitigated the financial impact of the lockdown for all recipients but that participants with disabilities still struggled more than others to ensure food security. These additional challenges may be related to pre-existing inequalities, with participants with disabilities and their households having less access to natural resources and financial stability.

**Contribution:**

This paper focuses on young women with and without disabilities and provides insight into the similarities and differences in their experiences.

## Introduction

The sustainable development goals (SDGs) of the 2030 agenda serve as a ‘shared blueprint’ and framework to guide countries and communities to achieve inclusive development for all (United Nations General Assembly 2015). Sustainable development goals 1 and 2 focus on ending poverty and hunger and include disaster management to ensure sustainable livelihoods for all (Our World in Data team 2023; United Nations Department of Economic and Social Affairs 2023). The SDG agenda’s pledge ‘to leave no one behind’ focusses on marginalised groups such as persons with disabilities. Disability-inclusive development requires participatory and inclusive approaches that include persons with disabilities as active participants and account for their abilities and vulnerabilities. Literature has established that persons with disabilities are more likely to live in poverty, experience food insecurities, and have higher opportunity and out-of-pocket costs than persons without disabilities (Banks & Polack 2015; Mitra, Posarac & Vick 2013; Mont et al. 2022; Quarmby & Pillay 2018; United Nations Department of Economic and Social Affairs 2018). The socio-economic inequalities affecting persons with disabilities are also gendered. Women with disabilities are more likely to experience poverty and food insecurity than men with disabilities (Mitra & Yap 2022; Schwartz, Buliung & Wilson 2019; UNFPA 2018; United Nations Department of Economic and Social Affairs 2018). In South Africa, persons with disabilities, particularly women with disabilities, earn less income and experience higher out-of-pocket costs (National Department of Social Development 2015). The country has implemented social protection mechanisms to counteract these inequalities, such as disability grants and employment equity policies that protect persons with disabilities (and other disadvantaged groups) (Hanass-Hancock & McKenzie 2017; Republic of South Africa 1998).

The recent coronavirus disease 2019 (COVID-19) pandemic devastated people’s livelihoods, depleting many of their assets. Reviewing the impact of the pandemic on SDGs, the International Labour Organisation (ILO) 2022 report on COVID-19 revealed that worldwide poverty rates had increased for the first time in two decades from 6.7% in 2019 to 7.2% in 2020 (ILO 2022). The report also revealed that unemployment had increased and was projected to stay above pre-pandemic levels until at least 2023; that youth unemployment was increasing and access to education or training was a persistent struggle; that the progress in addressing gender inequalities stalled and that small and informal businesses were disproportionally affected (ILO 2022). Hence, the pandemic depleted people’s assets and harmed their livelihoods and ability to meet basic needs such as food and shelter.

Globally, research revealed that disability-related inequalities widened during the COVID-19 pandemic (Banks et al. 2021; COVID-19 Disability Rights Monitor and Brennan 2020; Department of Women, Youth and Persons with Disabilities and United Nations Human Rights 2021; Mckinney et al. 2021; Rohwerder et al. 2021; Shaw et al. 2021; Thompson et al. 2021; Wickenden et al. 2021a, 2021b). Several of these publications describe the severe breakdown in providing support for persons with disabilities, who were without access to necessities such as food and nutrition and pushed further into poverty; some were even excluded from the pandemic response and social protection measures. In addition, they had limited access to COVID-19-related prevention and treatment information. This research also revealed that people with disabilities experienced significant barriers to accessing and receiving essential services such as healthcare.

Furthermore, survey analysis on food insecurity and disability during the pandemic in resource-rich settings revealed that households with persons with disabilities were more likely to experience food insecurities, were more impacted by the economic downturn and had higher levels of emotional distress (Brucker, Stott & Phillips 2021; Engelman et al. 2021; Friedman 2021; Sultana et al. 2023; Turk & Mitra 2021). In addition, women with disabilities, who were already more likely to experience social, economic and health disparities than men with disabilities or those without disabilities experienced even further inequality during the pandemic (COVID-19 Disability Rights Monitor and Brennan 2020; UNFPA 2021a). Therefore, the pandemic may have affected young women with disabilities disproportionally as they may experience multiple intersecting marginalisations based on gender, age, race and disability. However, very little empirical research focussed on youth with disabilities during the pandemic in low- or middle-income countries and even less on young women with disabilities. One of the few studies revealed that youth with disabilities experienced additional stigma and discrimination, struggled to maintain their livelihoods and felt excluded from government responses (Leonard Cheshire 2021). The UN 2020 report on the impact of COVID-19 on women and girls with disabilities and the Afrique Rehabilitation and Research Consultants (ARRC) report on the daily lives of adolescent girls and young women with disabilities both suggest that this group experienced barriers to accessing healthcare and education, including sexual reproductive health and rights (SRHR) (ARRC 2021; UNFPA 2021a). Despite this, we know little about how young women with disabilities experienced the pandemic, how it affected their livelihoods, how their experience differed from those without disabilities and whether protection mechanisms assisted them in navigating the crisis.

### South Africa

At the beginning of the pandemic in 2020, South Africa implemented one of the strictest global lockdown measures (with stay-at-home orders, closure of businesses and restrictions on ‘non-essential’ health services). This strict lockdown was followed by different lockdown levels implemented until March 2022 (Department of Women, Youth and Persons with Disabilities and United Nations Human Rights 2021). The country also implemented various social security measures, including a small COVID-19 social relief of distress cash transfer of ZAR350 (USD20), food parcels and tax breaks (Department of Women, Youth and Persons with Disabilities and United Nations Human Rights 2021). It also provided existing social grants, such as disability and child grants (Department of Women, Youth and Persons with Disabilities and United Nations Human Rights 2021). These measures speak to SDGs 1 and 2. They may have functioned as socio-economic protection mechanisms for persons with disabilities.

Concurrent with the COVID-19 pandemic, South Africa continued addressing its ongoing human immunodeficiency virus (HIV) epidemic, with new infections taking place, particularly among young women and those in KwaZulu-Natal (Burger et al. 2020). South Africa also implemented stringent lockdown levels, which can particularly impact young women in transitional stages of life who are completing or have just completed their tertiary education and training and come from poorer households. While many young women with and without disabilities may struggle to access tertiary education in South Africa, the country has implemented specific programmes to enable disadvantaged youth to access either universities, colleges or learnerships. These programmes include the provision of ‘free tertiary education’ through the National Student Financial Aid Scheme (NSFAS) for students from households earning less than ZAR350 000 (USD19 000) a year and learnership grants and stipends that act as a form of income. In addition, young women with disabilities can apply for a disability grant of ZAR 2090 (USD112), which is a monthly social protection cash transfer for South African citizens who earn under a specific threshold and have a medical certificate confirming disability status.

The Forgotten Agenda study was conducted to better understand the lived experiences of young women with disabilities during the pandemic and how they differed from women without disabilities of similar life trajectories as students or graduates in KwaZulu-Natal. The overall study not only focussed on disability and SRHR but also captured elements of livelihoods, as livelihoods are critical contextual factors that enable young people to claim their SRHR (UNFPA 2017, 2021a, 2021b). The study focussed on the province of KwaZulu-Natal, as it has one of the highest rates of new HIV infections and teenage pregnancies among girls and young women in the country (De Oliveira et al. 2017; South African National AIDS Council 2023).

This article focusses on the livelihood data from the Forgotten Agenda study. The availability of government social protection mechanisms allowed us to follow women with and without disabilities (18–25 years) through the pandemic and study if and how these mechanisms protected them and their households. This approach enabled us to understand the lived experience of participants who had access to social protection mechanisms and the impact of the COVID-19 pandemic on their livelihoods.

In this article, we use our participants’ narratives about their livelihoods. We aim to describe how young women with and without disabilities experienced the pandemic, how it affected their livelihoods and food security, and if and how their experience differs from those without disabilities. We will also discuss the potential use of these differences to inform progress towards those SDGs focussed on ending poverty and hunger.

## Research methods and design

A longitudinal participatory study was conducted with 72 young women in the eThekwini municipality of KwaZulu-Natal, a province in South Africa, during the COVID-19 pandemic. Data were collected from participants at multiple time points to cover the different lockdown periods between April 2020 and March 2022. During the COVID-19 pandemic, the study area underwent five distinct COVID-19 waves: a strict lockdown with firm stay-at-home orders from March to May 2020, followed by different lockdown levels. Additionally, severe civil unrest occurred in July 2021, leading to looting, destruction of businesses and 354 deaths (Department of Women, Youth and Persons with Disabilities and United Nations Human Rights 2021; Hunter, Singh & Wicks 2021). The overall study applied a pragmatic paradigm underpinned by social justice issues. It used a mixed methods approach, including quantitative, qualitative and photovoice methods to understand participants’ experiences during these turbulent times. This article presents the quantitative and qualitative results relating to participants’ livelihoods, including food security.

### Sample

**FIGURE 1:**
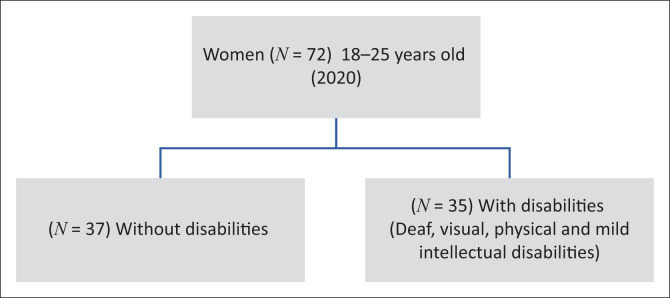
Sampling framework forgotten agenda study.

Purposive sampling was applied to ensure that women of similar age and educational background were recruited into the study. The study recruited 72 young women with and without disabilities who were aged 18–25 (in 2020); enrolled in or had completed tertiary education in eThekwini; willing to self-report on their experience of the lockdown and its impact on their lives; able to understand and provide informed consent; conversant in English, isiZulu or South African Sign Language (SASL); and willing to be audio or video recorded (video for ‘the Deaf’^1^). The final sample included 37 black women without disabilities and 35 black women who were deaf or had visual, mild intellectual or physical disabilities (Figure 1).

### Recruitment

The initial strict COVID-19 lockdown in South Africa made meeting people where they study, work, live or socialise impossible. Hence, we approached our first participants through online and mobile phone technology. The first 16 participants became seed participants. These participants were identified with the support of participants from our existing studies or through stakeholders at tertiary education facilities. Seed participants and tertiary education facilities then assisted in recruiting further participants of similar age and disability status.

Potential participants received an electronic flyer with a study summary from seed participants or tertiary facilities. After identifying interested participants, seed participants provided contact details to the study team, which screened them for eligibility before they were recruited for the study. This eligibility screening was conducted via phone or WhatsApp for all participants as this was the most feasible way to reach young people during the pandemic. This screening included oral or signed and, later, written consent. Disability status was established through self-identification and checked against the Washington Group Short Set on Functioning and an additional question prompting previous identification as a person with a disability (e.g. through going to a special school or receiving a disability grant). Eligible participants were invited to undergo the informed consent procedures and, after that, decided if they wanted to participate in the study. Informed consent procedures were conducted face-to-face for the Deaf to enable appropriate communication using SASL, while all other participants undertook informed consent remotely using phone and WhatsApp. Information about the study was provided verbally and in writing, using accessible formats that included pictures to overcome language barriers. Eligible participants who signed the informed consent (physically or electronically) were enrolled into the study.

### Data collection

The study used in-depth interviews conducted face-to-face with the Deaf and telephonically with all other participants. Participants were asked to identify a convenient and private place where they could freely express themselves. The interviewer checked this with the participant before the interview commenced. Face-to-face interviews were conducted for the Deaf at the eThekwini research office only after the strict lockdown ended and in alignment with lockdown regulations (e.g. shields, see-through dividing walls) (Banks et al. 2022). An interview guide was utilised, including a quantitative set of questions and open-ended questions prompting participants living and household situations, access to resources, food security, experience and worries about COVID-19, mental health, sexual reproductive health and rights, and exposure to violence. For food security, the Household Hunger Scale (HHS) was utilised (Ballard et al. 2011). The interview guide was made accessible through plain language and the usage of pictures and illustrations to include participants with intellectual disabilities and the Deaf. Illustrations were used to simplify complex concepts and overcome potential language and other access barriers. Interview guides were translated and back-translated by two research assistants fluent in isiZulu and English. Participants also received instructions on how to support their descriptions of their lived experience during COVID-19 with photos. This was carried out in a way that did not reveal the identity of the participants yet captured something related to their lived experience at the time of the interviews.

Interviews lasted approximately 1 h and were conducted in English, isiZulu or SASL. These interviews prompted the participant to retrospectively reflect on their situation before the lockdown, during the strict lockdown and during the other lockdown levels using two repeating interview waves (2020/21 and 2021/22). All interviews were audio or video (the Deaf) recorded and summaries were transcribed and translated into English.

### Data entry and analysis

The open-source suite for field data collection, KoboToolbox, was used to enter quantitative data (Kobo Inc 2022). Interviews were audio recorded, transcribed and translated into English by four research assistants controlling for quality through data entry, transcription and translation checks. The qualitative and quantitative data were cross-checked against each other to ensure consistency. If they differed (e.g. education status), fieldworkers directly verified the information with participants.

The software for statistical computing and graphics ‘R’ was used to analyse the quantitative data (R Core Team 2022). Descriptive statistics were developed for all questions and the HHS and disaggregated for women with and without disabilities to assess differences. The descriptive tables use means and standard deviations for continuous variables and percentages for categorical variables. The HHS score uses a median and interquartile range (IQR) per developer recommendations (Ballard et al. 2011).

In addition to the median, the HHS is summarised using a categorical indicator. The HHS score was calculated by recoding each question so that an answer of ‘No’ or ‘None’ scored 0, an answer of ‘Rarely’ or ‘Sometimes’ scored 1 and an answer of ‘Often’ scored 2. The three questions were added to get a score between 0 and 6. This score is generally not normally distributed, and it is thus recommended to summarise it using a median rather than a mean. This score is also categorised for the HHS categorical indicator, with a score of 0–1 indicating ‘Little to no hunger in the household’, a score of 2–3 indicating ‘Moderate hunger in the household’ and a score of 4–6 indicating ‘Severe hunger in the household’.

Significance testing used Fisher’s exact test for categorical variables, *t*-tests for continuous variables and the Kruskal–Wallis Rank Sum Test for the HHS score. Three significance tests were run for each variable, looking at differences between women with and without disabilities at each time point. *p* values greater than 0.05 were considered to be significant.

The information from each participant’s qualitative and quantitative data and photovoice were combined to develop a case study for each participant. The qualitative and quantitative data were entered and analysed using guided content analysis and the qualitative analysis software NVIVO (version 12) and were reported here. After familiarising themselves with the data, the researchers identified common themes and developed a codebook. After that, two independent researchers coded each case study using this codebook. Codes were compared and differences were addressed between the coders through discussions leading to agreement. Theme summaries were developed for each emerging theme and its sub-themes. Nine major themes emerged, of which three (‘Impact of the pandemic on livelihoods’, ‘Capabilities’ and ‘Food security’) were found to be relevant to livelihood experiences and are the focus of this article.

### Ethical considerations

The study received ethical clearance from the South African Medical Research Council Ethics Board (Reference numbers: EC020-6/2020 and EC001-2/2021). All participants underwent informed consent procedures and signed the informed consent form. The informed consent explained the study’s purpose, procedures, risks and benefits. It also clarified the voluntary nature of the study and confidentiality measures and provided contact details. Participants who needed further support or counselling had access to the study nurse and counsellor, who referred participants to any additional services if needed.

Collected data were saved on the organisation’s shared drive and password-protected computers only accessible to study staff.

The research team comprised of the principal investigator, co-investigator, study coordinator and research assistants. It consisted entirely of South African-based researchers, including white, black and Indian staff of different genders. The fieldwork team included South African women with and without disabilities; two spoke fluent isiZulu and English, and one was also fluent in SASL. The team included two researchers with a disability. Hence, the research team included a diverse staff, encompassing participants of different races, ages, genders and disability statuses. Most team members were highly experienced research staff with prior qualitative research experience and training. One team member had comprehensive training, experiences and skills needed to adapt educational and information material to research questionnaires and methods. One team member was trained for the first time and supervised by the other staff members. A validation meeting with participants (5) and stakeholders (5) from the tertiary institutions and the Department of Health (provincial and national) provided feedback to the community and confirmed study results and gaps.

## Results

Overall, we recruited 72 young women (aged 18–25 in 2020) into our study, of which 37 were women without disabilities and 35 had different kinds of disabilities (Deaf, visual, physical and mild intellectual disability). Most participants (89%) were students in 2020 (Table 1), while the rest graduated. They had enrolled in mainstream universities, technical vocational education and training (TVET) colleges or specialised colleges that accommodated the needs of young persons with disabilities (Table 1). The latter specifically provided education for women with intellectual disabilities and the Deaf. As such, participants with and without disabilities managed similar life trajectories as students or new graduates during the COVID-19 pandemic.

**TABLE 1:** Demographics and living arrangements.

Variables	Pre-COVID-19				Strict lockdown				Soft lockdown			
	Women without disability (*n* = 37)		Women with disability (*n* = 35)		Women without disability (*n* = 37)		Women with disability (*n* = 35)		Women without disability (*n* = 29)		Women with disability (*n* = 27)	
	Mean	s.d.	Mean	s.d.	Mean	s.d.	Mean	s.d.	Mean	s.d.	Mean	s.d.
Age in 2020	22.38	2.52	22.00	2.00	-	-	-	-	-	-	-	-
Number of people living in residence	3.35	2.29	6.69	8.91	5.97	2.87	6.26	2.62	5.52	3.71	4.81	2.94

s.d., Standard deviation.

### Livelihoods

In our study, participants explained how they managed their livelihoods during the COVID-19 pandemic. Participants with and without disabilities had similar challenges related to their living spaces, continued education and household income stability (physical, human and financial capital). Participants without disabilities also discussed how they built gardens to plant crops and support the family (natural capital). They discussed what necessities they could acquire such as food, water, shelter and clothing and what resources they and their families accessed to make a living. These resources or capitals included their housing and living spaces, availability of land, income (earned, stipends and grants), and social connectedness and support. These resources or capitals are related to how participants and their families maintained their livelihoods and ensured food security. Participants with disabilities did not report accessing natural resources (e.g. planting crops), and some struggled more regarding their social connectedness and ability to secure necessities such as food.

### ‘Learning about each other’ – Housing and living spaces during the pandemic

One of the first significant changes experienced by participants was related to the initial strict lockdown in March 2020. Participants and their family members had to return to their primary family homes, which often increased household sizes. Before the COVID-19 pandemic, participants without disabilities were more likely to live in a student residence (sharing spaces with fewer young people) than participants with disabilities (Table 2). This dynamic changed during the strict lockdown period (March–May 2020), where most participants, regardless of disability status, lived with their families and could leave the house only for essential activities such as ‘grocery shopping’ or other essential services. Most of these family homes were in rural areas or freestanding houses in townships (historically established suburbs commonly referred to as underdeveloped, urban residential areas formerly officially designated for ‘non-white’ people by apartheid legislation) (Li & Godehart 2007). During the strict lockdown, participants and their family members had to work or study from home if their work was not on the ‘essential work’ list. Participants and their families had to learn how to live, work and study in a household with many family members. This change in living arrangements continued over the softer periods of lockdown, where family members gradually returned to work or studies at different times.

**TABLE 2:** Demographics and living arrangements.

Variables	Pre-COVID-19				Strict lockdown				Soft lockdown			
	Women without disability (*n* = 37)		Women with disability (*n* = 35)		Women without disability (*n* = 37)		Women with disability (*n* = 35)		Women without disability (*n* = 29)		Women with disability (*n* = 27)	
	*n*	%	*n*	%	*n*	%	*n*	%	*n*	%	*n*	%
Student in 2020	31	83.8	33	94.0	-	-	-	-	-	-	-	-
**Type of residence** †												
Separate dwelling	14	37.8	27	77.1	34	91.9	33	94.3	25	86.2	25	92.6
Student residence	22	59.5	7	20.0	0	0.0	1	2.9	2	6.9	2	7.4
Backyard dwelling	1	2.7	1	2.9	3	8.1	1	2.9	2	6.9	0	0.0

† , Significant difference between women with and without disabilities pre-COVID-19.

Overall, participants without disabilities described mixed experiences when reflecting on their changed living spaces. While these participants missed their freedom and independence living in the student residences, they appreciated the togetherness and time spent with their families:

‘It was a very emotional period … I can’t go out and do what I normally do, go see my friends and go shopping … we [family members] understand each other, and we are able to help each other, emotionally and physically.’ (23-year-old, woman, without disability)

Similarly, the experiences of participants with disabilities varied depending on their situation before the lockdown. Some participants were living with family members in eThekwini – the town of their tertiary education institution. These participants had other family members join the household during the strict lockdown and spent time getting to know these family members:

‘During Covid-19, we were just bonding with my family and …, we did learn about each other.’ (23-year-old, woman, with intellectual disability)

Other participants with disabilities had been staying far away from their families as eThekwini provides some of the few accessible tertiary institutions geared to accommodate the needs of the Deaf or those with intellectual disabilities. These participants would move to their families for the strict lockdown and then back into town when these specialised educational institutions reopened. Specialised educational institutions were allowed to reopen for face-to-face interactions earlier than other institutions. As a result, these participants could interact with friends and staff earlier than participants registered in mainstream tertiary institutions.

### ‘Finishing this degree’ – Continued education during the pandemic

At the beginning of the COVID-19 pandemic, most participants were students. During the strict lockdown, they had to adjust from in-person to online academic learning. Participants with and without disabilities reported similar challenges, including noisy environments, a lack of Internet connectivity or data, and space to study comfortably:

‘The connection [internet] is bad and … we are living together with the family and most of the time I have to do the chores of the house and when I am trying to study the kids are disturbing …’ (19-year-old, woman, without disability)

‘I do not like a lot of noise, when I tried to study, they [family members] kept making noise.’ (20-year-old, blind woman)

Participants reported taking over additional responsibilities to manage the household. These responsibilities generally included cleaning, cooking and looking after the children. Participants with and without disabilities expressed concerns about continuing their studies and the effects on their future. Anxieties were heightened for participants with disabilities who had experienced challenges in studying before the pandemic. Family and friends were crucial social support structures:

‘I talk to friends and people close to me when I am feeling sad and lonely. The thought of the future and not being employed depressed me …’ (23-year-old, woman, without disability)

‘I was worried about my future … when “Corona” came, I said there is no way I will finish this degree. I was devastated; I use to cry at night. I would call my mother and say: “you know, today I feel like taking my life”, then she would motivate me and uplift my spirit.’ (25-year-old, woman, with physical disability)

The challenges in accessing and completing education changed for participants depending on their tertiary institution. Participants with disabilities at institutions targeting young persons with disabilities (e.g. learnership programmes and colleges for young persons with disabilities) had their educational challenges mitigated earlier as their institutions were allowed to return to face-to-face lectures months before mainstream universities opened for in-person lectures:

‘I am back and happy to study IT at [Institute for the Deaf]. I see my friends there, and I am not lonely.’ (24-year-old, Deaf woman)

Hence, challenges to continuing their studies existed for participants with and without disabilities. These challenges included noisy or crowded homes, Internet connection challenges and additional workload managing the household. Adjusted lockdown regulations enabled tertiary institutions focussing on young persons with disabilities to respond faster to students’ educational needs than their mainstream counterparts.

### ‘Using grants to buy food’ – Earning a living during the pandemic

During the strict lockdown, only essential services were allowed to continue operating face-to-face. People outside these services needed to work or study from home. People lost their jobs or had reduced income, where it was not possible to work from home. Participants with and without disabilities reported on family members losing their jobs, being retrenched, or being unable to earn an income with their informal businesses. The households of participants with and without disabilities lost income. Many survived on social grants (old-age pension, child support and disability) or student stipends:

‘Many people were retrenched and lost their job. Uhhm, the food prices went sky up high, making it difficult for people who are earning less salary and those who are not earning at all to be food secure.’ (21-year-old, woman, without disability)

‘We had to use my granny’s grant and my grant to buy food because I had to be supportive since I wasn’t going to school.’ (20-year-old, blind woman)

This situation improved in some households after the strict lockdown levels were lifted and people could return to work:

‘Now [soft lockdown] many of my aunties are going to work, they can afford the groceries, everyone is contributing towards the groceries.’ (18-year-old, blind woman)

Student stipends and scholarships were important sources of income for participants with and without disabilities and their families. Most students without disabilities accessed the National Student Financial Aid Scheme (NSFAS) and received a monthly living allowance of approximately ZAR1400/USD80 (+ housing and material support). Those students in colleges providing training and learnerships for young persons with disabilities received a stipend of approximately ZAR3000/USD167 for living and housing. If participants with disabilities did not attend their tertiary studies, they could no longer receive the college-specific stipend but could access the disability grant of approximately ZAR1900/USD106 (one cannot receive the disability grant and the stipend). A few participants reported their families accessing the COVID-19 disaster grant or food parcels. Hence, this sample of participants could access social protection schemes and student support.

### ‘Making a garden’ – Using land and gardens to plant crops

Those participants who returned from student residencies to their family homes reported moving back into rural areas with larger homesteads or freestanding houses in townships. Participants moving back to rural areas reflected on the change in their lifestyle, with many supporting the acquisition of water or firewood and planting of crops. These reports were mainly made by participants without disabilities:

‘I had to go back collecting water, wood here in [name of rural area]. Life has reversed from being a student to coming home.’ (19-year-old, woman, without disability)

Many participants without disabilities reported that their families used their land to plant crops for their usage and food security. These participants reflected positively and sometimes with pride on their experience of growing vegetables and bonding with other family members:

‘I knew that in some cases, we will end up not having enough money to provide for people, so I thought I should make a garden for myself so that I just have veggies and for healthy, balance diet. I needed veggies for me, my dad and mom.’ (18-year-old, woman, without disability)

Following these participants through the pandemic revealed that for some, the new activities of planting and harvesting crops and products supplied their households with fresh food and became a source of income by selling crops and other goods to community members:

‘I started a project of planting crops and selling them to neighbours, so that I can make extra cash. The aim is to have a bigger garden and be able to supply some stores.’ (19-year-old, woman, without disability)

Similar reports about gardening, planting crops or starting income-generating activities were not made in the group of women with disabilities, suggesting that they did not participate in these activities or that these families did not have access to land and livestock.

### ‘Communicating with my family’ – Staying socially connected during the pandemic

All participants lived with their families during the strict lockdown, and for many participants, this reality held for several months. Depending on their universities’ regulations, participants returned to their student accommodation at different times. The strict stay-at-home orders and enforced move to family homes separated most participants physically from their friends and broader social network while bringing them close to their families. Participants experienced this differently. While participants reported that they missed their friends and tried to stay connected using social media, some experienced the time with their families positively:

‘I’d call [WhatsApp] my friends and tell them whatever is going on at that moment, … if my problems are not secrets, then I’d just tell my mom, [*M*]om. I’m going through something like this, and she will sit me down and give me advice …’ (19-year-old, woman, without disability)

Participants’ experiences depended on the ability to communicate and interact with family members. The Deaf experienced communication challenges with their family members as their families did not know SASL. These participants had attended special schools for most of their schooling. These schools are far from their families, and while participants learned SASL, their family members did not. In addition, these schools use English as a medium of instruction, while the Deaf participants’ family members speak isiZulu. This reality made lipreading impossible as an alternative. As a result, the Deaf experienced the strict lockdown with their families as isolating as they could not communicate with their family members properly:

‘I couldn’t communicate with my family as they don’t know sign language, only basic/common signs. I was the only deaf person there, it was very hard and lonely.’ (25-year-old, Deaf woman)

Participants with disabilities also discussed factors that assisted them in keeping socially connected. Family members’ interest in learning augmented communication (e.g. SASL), sign language in news channels, accessible living spaces, access to the Internet and support were essential factors that made participants feel cared for and included:

‘I studied at home and taught my little nephew how to sign. My family did basic signs to me. I was lonely, but my little nephew kept me busy.’ (23-year-old, Deaf woman)

‘I have a strong support system. It allows me to be myself, allows me to be comfortable in my own skin … Everything is easily accessible; people here make it easy.’ (25-year-old, woman, with physical disability)

‘There was an interpreter on the TV signing. I was impressed because I could follow the news.’ (21-year-old, Deaf woman)

Hence, the accessibility and inclusion levels in the home environment determined if participants with disabilities could stay connected during a crisis. These accessibility and inclusion measures were established before the COVID-19 pandemic and determined how the participants felt socially included during the pandemic.

### ‘Buying food’ – Capabilities to meet basic necessities during the pandemic

Participants reflected on their capabilities to acquire essential goods for their livelihoods during the pandemic. Those with and without disabilities explained that they supported their families financially with their student stipends and social grants. Participants without disabilities also reported on planting crops. Participants’ human and financial resources were used to provide food for themselves and their families; support building house structures; and pay for transport, Internet data and other commodities (e.g. menstrual products). Some participants with disabilities also reflected on ‘sending money’ to other family members not living with them, either to support these family members or to look after their children:

‘My sister and I get NSFAS and the social grant (child grant) … we combined money and bought lots of food at the beginning of the month so that the food would last us longer. And those who are working contributed … and my grandmother contributed so we would have food that would last us longer.’ (20-year-old, woman, without disability)

‘Once a month, I buy a big bag of mealie meal [maize meal]. I will eat chicken, meat and veg when I have enough money. I struggle with money. I pay R1200 to my Aunty, who looks after my daughter.’ (25-year-old, Deaf woman)

Earned income, stipends and grants were critical financial resources for families. Hence, households with employed family members managed better through the pandemic. Participants often shared part of their stipends with the family to buy food, focussing on buying essential groceries:

‘She [mother] used to buy groceries at the beginning of the month … when we [siblings] get our allowances [stipend], we would contribute R500 in the middle of the month to go and buy more groceries, so in that way, we managed to have enough food and not starve.’ (21-year-old, woman, without disability)

‘We used my grandmother’s grant and my disability grant to buy food once a month.’ (20-year-old, woman, with physical disability)

Hence, depending on their household situation, participants spent their stipends on themselves or shared it with others. Participants who could stay by themselves and did not have family responsibilities were better off. For instance, one blind participant described that she could return to her student residence after the strict lockdown and buy her own groceries and goods:

‘I received an allowance [student grant] on a monthly basis, I stay by myself … I buy what I need in that month.’ (21-year-old, blind woman)

Other participants with disabilities said they struggled with money even if they had returned to their study arrangements. These participants had to share their student stipends with other family members, providing for their own children or family members who did not earn any income. These participants indicated that they struggled with their available finances:

‘My siblings don’t have jobs, they always ask for money for food, and they also say my child is sick [siblings look after the child].’ (24-year-old, Deaf woman)

Hence, depending on the family situation, participants experienced challenges acquiring essential goods. Some participants with disabilities struggled more to ensure enough resources were available to meet their own and their family’s needs.

### ‘Not eating all the time’ – Impact of the pandemic on food security and hunger

Participants with and without disabilities experienced similar financial situations as both groups had access to financial aid through student stipends and experienced additional financial responsibilities to support their households. However, participants with disabilities were more likely to report food insecurity and hunger. Overall, participants without disabilities less frequently reported challenges with accessing enough food than participants with disabilities, and this difference held throughout the pandemic. This trend was observed in all the questions in the HHS, namely that they had no food in the house, went to bed hungry or had nothing to eat all day (Table 3). The difference was still significant in the soft lockdown phase, suggesting a persistence of long-term inequalities. In addition, participants with disabilities more frequently reported accessing food parcels and social grants (child support, disability and COVID-19 disaster grants).

**TABLE 3:** Food security and social protection.

Variables: In the past 4 weeks	Pre-COVID-19				Strict lockdown				Soft lockdown				*p*
	Women without disability (*n* = 37)		Women with disability (*n* = 35)		Women without disability (*n* = 37)		Women with disability (*n* = 35)		Women without disability (*n* = 29)		Women with disability (*n* = 27)		
	*n*	%	*n*	%	*n*	%	*n*	%	*n*	%	*n*	%	
No food to eat of any kind (at least rarely) = Yes	-	-	-	-	13	35.1	15	42.9	2	6.9	14	51.9	§
Gone to sleep at night hungry (at least rarely) = Yes	-	-	-	-	3	8.1	7	20.0	2	6.9	6	22.2	-
Gone whole day and night without eating (at least rarely) = Yes	-	-	-	-	0	0.0	4	11.4	1	3.4	6	22.2	§
Accessed Food parcels = Yes	2	5.4	4	11.4	8	21.6	12	34.3	2	6.9	6	22.2	-
Accessed Social or COVID-19 disaster grants = Yes	1	2.7	14	40.0	14	37.8	20	57.1	8	27.6	18	66.7	†,§

† , Significant difference between women with and without disabilities pre-COVID-19.

‡ , Significant difference between women with and without disabilities during strict lockdown.

§ , Significant difference between women with and without disabilities during soft lockdown.

Similarly, the overall hunger score indicated that participants with disabilities were more likely to experience moderate hunger in their households. This difference held throughout the pandemic, with the gap widening as the pandemic progressed (Table 4).

**TABLE 4:** Household Hunger Scale.

Variables	Pre-COVID-19								Strict lockdown								Soft lockdown								*p*
	Women without disability (*n* = 37)				Women with disability (*n* = 35)				Women without disability (*n* = 37)				Women with disability (*n* = 35)				Women without disability (*n* = 29)				Women with disability (*n* = 27)				
	*n*	%	Median	IQR	*n*	%	Median	IQR	*n*	%	Median	IQR	*n*	%	Median	IQR	*n*	%	Median	IQR	*n*	%	Median	IQR	
Categorical HHS Indicator	-	-	-	-	-	-	-	-	-	-	-	-	-	-	-	-	-	-	-	-	-	-	-	-	‡, §
Little to no hunger in the household (0–1)	-	-	-	-	-	-	-	-	34	91.9	-	-	24	68.6	-	-	27	93.1	-	-	19	70.4	-	-	-
Moderate hunger in the household (2–3)	-	-	-	-	-	-	-	-	3	8.1	-	-	10	28.6	-	-	2	6.9	-	-	8	29.6	-	-	-
Severe hunger in the household (4–6)	-	-	-	-	-	-	-	-	0	0.0	-	-	1	2.9	-	-	0	0.0	-	-	0	0.0	-	-	-
Median HHS score	-	-	-	-	-	-	-	-	-	-	0	0, 1	-	-	0	0, 2	-	-	0	0, 0	-	-	1	0, 2	§

HHS, Household Hunger Scale; IQR, Interquartile range.

† , Significant difference between women with and without disabilities pre-COVID-19.

‡ , Significant difference between women with and without disabilities during strict lockdown.

§ , Significant difference between women with and without disabilities during soft lockdown.

Participants with and without disabilities reported heightened awareness in their families to manage food carefully. Participants without disabilities reported that their families would opt to buy food in bulk and supplement their family meals with the crops gained from their gardens:

‘We pile stock every beginning of the month on basic needs that are going to be used during the month … we limited how often we eat in a day.’ (19-year-old, woman, without disability)

‘Vegetables were harvested at the home garden and were not bought from the shop. The garden kept a vital role at home.’ (25-year-old, woman, without disability)

Furthermore, participants without disabilities reflected on the contribution of family members who were working or receiving some form of social grant. These participants described how they contributed to food security with their student stipend to counteract the loss of other family members’ income during the strict lockdown and that they could stop these contributions once they were back at work:

‘With my NSFAS money, I used to buy groceries [strict lockdown] … the food situation is much better now [soft lockdown] because our brother provides more money.’ (19-year-old, woman, without disability)

‘My aunt works at Illovo, she is a breadwinner, buys food and gives us money every month. Grandmother’s money buys something, but only if something in the household is short.’ (20-year-old, woman, without disability)

Overall, participants without disabilities seldom reported food insecurities. While some reported adjusting their food sources to cheaper options, others reflected on overeating during the strict lockdown, as family meals were among the few forms of entertainment left to people. In these families, eating habits normalised after the strict lockdown:

‘We are now back to buying once a month and have now improved, we don’t eat all the time as before [during the strict lockdown] because people are back at work and school because boredom is what made us eat a lot [during the strict lockdown].’ (19-year-old, woman, without disability)

Participants with disabilities also reported that their families would opt to buy food in bulk, manage their resources carefully and use leftover food. Only one participant with disability reported that their family used a garden to grow crops. Overall, participants with disabilities described that ensuring enough food was available for their families was hard. Some also reported that their family relied on them for financial support to acquire food for the household:

‘At home, we buy food at the end of every month because that is when my mother and I get paid, we make use of the grant money from my late sister’s children and the money my mother and I get paid to contribute towards the groceries at home, it is a lot of groceries, and we all know that it will last us.’ (23-year-old, woman, with intellectual disability)

‘We have nine adults and some young children. Some of my siblings are not working. My mother relies on me for money because I get money from studying. My siblings don’t help enough. My mother is old and it is hard on me … Sometimes I only eat breakfast and skip meals as I struggle to have enough money.’ (24-year-old, Deaf woman)

Some participants with disabilities also described that their families rationed portions or skipped meals. These participants reported changing their routine in eating habits or reducing the number of meals their families would have. Some participants reported going to bed hungry, as they lacked the resources to buy food:

‘Ever since lockdown started, we changed the routine of eating, we normally eat three times a day, so we changed it to two times a day, breakfast and lunch was combined, and then we eat supper at eight … We used my grandmother’s grant and my disability grant to buy food once a month.’ (29-year-old, woman, with physical disability)

‘I eat mostly breakfast, miss lunch, and then eat supper. Sometimes I go to bed hungry because I don’t have enough money to buy food.’ (25-year-old, Deaf woman)

Hence, despite the similarity of life situations among our participants with and without disabilities and their similarities in access to financial aid and student support, participants with disabilities more frequently reported times of food insecurity or hunger in their households.

## Discussion

Our study followed a cohort of young women with and without disabilities in eThekwini, KwaZulu-Natal, South Africa, who were enrolled in (or had just completed) tertiary education through the COVID-19 pandemic. While all participants experienced similar challenges during the pandemic, their experiences of food security and coping mechanisms were different. Literature on food security and disability is emerging (Mitra 2013; Mitra et al. 2013; Schwartz et al. 2019; United Nations Department of Economic and Social Affairs 2018). Existing evidence shows that persons with disabilities are more likely to experience poverty and food insecurity, as poverty and food insecurity are intertwined. While the evidence on food security shows a causal relationship between disability and food insecurity, it does not yet consider the broader social and environmental context (Schwartz et al. 2019). Schwartz et al.’s literature review of 106 articles covering disability and access to food or food (in)security worldwide showed that the mediators of the relationship between disability and food insecurity are underexplored. The authors state that the literature mainly conceptualises disability as a factor preventing access to food while neglecting disabling social and environmental barriers (Schwartz et al. 2019). Our study suggests that pre-existing inequalities (e.g. access to natural and social capital) are potentially such mediators even when participants’ life trajectories are similar. Schwartz et al. review also revealed that literature is mainly available from resource-rich countries (only one study was from Africa, specifically Ethiopia), focussed on older people (only four studies were on the group between 18 and 25 years of age), and that studies compared people with and without disabilities without considering their specific life situations in terms of access to livelihood capitals, support and inclusion (Schwartz et al. 2019).

Our study provides a unique opportunity to compare the experiences of young women with and without disabilities in similar life trajectories as students or graduates during a crisis. The data provide insight into the complexities of livelihood capitals, support and inclusion, and how these are connected to the participants managing scarce resources and food security during a crisis.

Participants’ situations were similar regarding their living spaces, their need to further their education during the COVID-19 pandemic, their ability to access student financial support schemes and social grants, and their additional responsibilities to manage the family’s housework. The latter is likely linked to our sample being young women. As such, cultural or gender expectations dictate that they support cleaning, cooking and looking after children. These expectations applied to both women with and without disabilities.

However, despite the similar life trajectories in education and socio-economic contexts, participants with disabilities were more likely to experience food insecurities in their households, and this seems to be related to lower livelihood capital in the participants’ households. The literature recognises reduced financial resources, higher expenses and reduced coping mechanisms (bargain hunting, home cooking, gardening, social assistance, etc.) as key factors explaining the food insecurity-disability nexus (Heflin, Corcoran & Siefert 2007; Huang, Guo & Kim 2010; Schwartz et al. 2019). Geographical factors (e.g. availability of shops vs. ‘food deserts’); biological factors; environmental barriers; and social norms, attitudes and support are also identified as mitigating factors that enable or reduce access to affordable food sources (Schwartz et al. 2019).

Our study showed that participants with and without disabilities had access to student stipends and social grants (financial capital). These financial support mechanisms became essential resources for the participants’ entire households during the COVID-19 pandemic. Participants’ households experienced similar challenges in living in larger households, with family members’ income loss and with enforced lockdown restrictions. Considering that depending on the source, between 7% and 20% of South Africans live below the international poverty line, it is unsurprising that the participants’ financial resources became essential to families in times of crisis (Our World in Data team 2023; United Nations Department of Economic and Social Affairs 2023).

However, the participants’ households dealt with the pandemic differently. While all participants experienced challenges and heightened awareness to ensure food security, households of participants without disabilities reported utilising land and gardens to plant crops and substitute food sources from subsistence farming activities (natural capital). Only one participant with a disability mentioned ‘gardening’ as an activity (by other household members) to provide food for the household. Despite this additional food source, participants with disabilities were more likely to report challenges with accessing food throughout the pandemic, compared to participants without disabilities. Participants with disabilities described their student stipends (and, alternatively, disability grants) as important sources of income for their families, which they shared to provide for themselves, other family members and their own children. The qualitative data suggest that participants with disabilities and their families had less access to financial and natural resources (income and land) overall. Local and international literature on poverty and disability reveals that both persons with disabilities and their households are more likely to experience unemployment and poverty and that this is also gendered (Banks & Polack 2015; Hanass-Hancock et al. 2017; Mitra & Yap 2022; Mitra et al. 2013; National Department of Social Development 2015). Furthermore, literature on land ownership reveals that persons with disabilities and their households are less likely to own and utilise land (United Nations Department of Economic and Social Affairs 2018). Our findings seem to align with this reality.

The 2018 UN Flagship report revealed that persons with disabilities are more likely to live in food-insecure households (United Nations Department of Economic and Social Affairs 2018). This reality continues to be the case in research on persons with disabilities conducted during the COVID-19 pandemic (COVID-19 Disability Rights Monitor & Brennan 2020; Mckinney et al. 2021; Wickenden et al. 2021b). Our study adds to this body of literature focussing on young women with disabilities in a middle-income country (South Africa) and how their experience compares to young women without disabilities. The participants’ experiences were similar regarding their living spaces, initial educational challenges and added responsibilities to support the housework. Specialised educational institutions were able to respond faster to the needs of young persons with disabilities. Differences appeared regarding financial management and food security, which calls for reconsidering the purpose of social grants targeting persons with disabilities. Social protection mechanisms such as the disability grant in South Africa are linked to available income. Thus, they are poverty-mitigating grants, not measures to address disability-related inequalities (Hanass-Hancock & McKenzie 2017; National Department of Social Development 2015).

Reconceptualising the purpose of disability grants as a mechanism to counteract inequalities will help to mitigate poverty further and treat persons with disabilities as equal members of society who are also providers for their families. For instance, if means testing for the disability grant is removed, the grant would be allocated based on disability status alone. Persons with disabilities could then access the disability grant regardless of their source of income. This change will assist them with the extra opportunity and out-of-pocket costs associated with disability, as these costs make it more difficult for them to provide for their families (Hanass-Hancock & McKenzie 2017; Hanass-Hancock et al. 2017; National Department of Social Development 2015). Such a reconceptualisation will also affect our understanding of the international poverty lines set out in the SGDs, which need to be higher for persons with disabilities and their households (SDG 1). Similarly, cash benefits (grants) targeting disability inequality would need to be tracked separately, targeting all persons with disabilities with social protection mechanisms to mitigate disability-related inequality (SDG 1). SDG 1 and 2 indicators also need to be disaggregated by disability and gender to understand if persons with disabilities experience poverty and hunger and, if so, which group of persons with disabilities is most affected. This calls for disability-disaggregated data collection, monitoring and evaluation of progress towards the SDGs.

Our study also revealed essential social and environmental factors that affect crisis management. Participants with disabilities identified social support, home environment accessibility, Internet access and communication accommodation as essential factors that helped them to cope during the pandemic or, in their absence, feel excluded. Hence, this experience emphasises the importance of enabling households with persons with disabilities to develop accessible home environments (including assistive devices) and communication methods before a crisis occurs. Approaches that considered the specific needs of young persons with disabilities, such as returning students with disabilities to face-to-face lectures and providing accommodation, were critical to crisis management. While this worked for some of our participants, literature worldwide suggests that pandemic responses with social distancing regulations often neglected the needs of persons with disabilities, in particular young persons and women with disabilities (COVID-19 Disability Rights Monitor and Brennan, 2020; Leonard Cheshire 2021; UNFPA 2021a). Our study shows that it is possible to respond differently and, through this, mitigate some of the impacts of a crisis. However, this was only possible where social protection was accessible, where colleges or universities could adjust quickly to the needs of young persons with disabilities, and where young persons with disabilities had access to the necessary disability accommodation in their home and study environment. Hence, pre-existing inequalities and access to mitigating mechanisms (e.g. accessible education and social protection) are vital to ensuring that people with disabilities are not left behind in times of crisis. While adapting the disability grant mitigates some of these inequalities, mainstreaming disability across social protection and critical programmes and policies is another way to create more equitable societies. This mainstreaming must be monitored with disability-inclusive indicators across programmes, including poverty alleviation and food security.

### Limitations

The study provided rich information about the experiences of young women studying in eThekwini, KwaZulu-Natal, South Africa with particular insight into the similarities and differences of their experiences depending on their disability status. The study is limited to a small group (72 participants) and included tertiary-level students and graduates who predominately had access to government financial aid. While this approach enabled a focus on differences based on disability (isolating the intersection with gender or socio-economic status), it limits the representativeness of the study to this group. Hence, the study does not represent the experience of young people in South Africa overall. In addition, the fieldwork was conducted during the COVID-19 pandemic, and alternative approaches to data collection were applied, namely, social media (WhatsApp) and phone conversations with most participants. This approach enabled broader inclusion of participants but could have limited how much participants shared with researchers (Banks et al. 2022).

As the original study focussed on sexual reproductive health and rights and disability, it did not include comprehensive data on livelihood, poverty and food insecurity. While the available data provided much-needed insight in a unique context, more information is needed to understand the disability-food-security nexus fully. More nuanced research is needed to understand livelihood differences between persons with and without disabilities and how these intersect with different life circumstances, gender and age.

## Conclusion

This study shows that social protection mechanisms mitigated the impact of the COVID-19-related lockdown for all recipients and their households in times of crisis but that households of participants with disabilities still struggled more than others.

Participants with and without disabilities reported similar experiences related to their housing, education and financial situations (physical, human and financial capital). They also reported similar gendered responsibilities during the pandemic including housework and having to look after children, while trying to keep engaged with their studies.

While all participants describe changes in how and with whom they could socialise and gain support, Deaf participants described experiencing significant social isolation (low social capital). Similarly, while participants without disabilities elaborated on utilising gardens, none of the participants with disabilities reported on the usage of gardens or crops to mitigate the impact of lockdowns on food security (natural capital). Overall, participants with disabilities reported more challenges in utilising their resources (capital) to buy necessities for their daily lives (capabilities) and more food insecurity or hunger in their households. These additional challenges may be related to pre-existing inequalities, with participants with disabilities and their households having less access to natural resources and long-term financial stability, even if they come from similar backgrounds and follow similar life trajectories.

## Footnotes

1 The article uses ‘the Deaf’ when speaking about participants who are deaf and use SASL as their main language and prefer to identify as the Deaf (National Institute for the Deaf 2022; Stander and Mcilroy 2017).
